# Is the *v*Lamax for Glycolysis What the $\dot{V}{\mathrm{O}}_{{2}}$ max is for Oxidative Phosphorylation?

**DOI:** 10.1007/s40279-025-02259-6

**Published:** 2025-07-17

**Authors:** Henning Wackerhage, Athanasios Kabasakalis, Stephen Seiler, Hermann Heck

**Affiliations:** 1 Exercise Biology, School of Medicine and Health, Technical University of Munich, Munich, Germany; 2 Laboratory of Evaluation of Human Biological Performance, School of Physical Education and Sport Science, Aristotle University of Thessaloniki, Thessaloniki, Greece; 3 Department of Sport Science and Physical Education, University of Agder, Kristiansand, Norway; 4 Emeritus Professor, Sports Medicine, Ruhr University Bochum, Bochum, Germany

## Abstract

Oxidative phosphorylation and glycolysis are the two truly major energy metabolism pathways in humans. While maximal oxygen uptake ($\dot{V}{\mathrm{O}}_{{2}}$ max) has been used for a century as a whole-body measure of maximal oxidative phosphorylation, there is no universally accepted, comparable measure of maximal glycolysis. However, already in 1984, Alois Mader introduced the maximal rate of lactate accumulation in mmol/kg/s related to active muscle weight (*v*Lamax_muscle_) for his mathematical model of human exercise metabolism. In 1994, on the basis of a critical analysis of glycolytic tests at the time, Mader proposed a practical test of the maximal rate of lactate accumulation in mmol per litre of earlobe or fingertip blood per second, corrected for alactic time (*t*_alac_), that is measured during an ~ 10–15-s all-out exercise test (*v*Lamax_blood_). The variant *v*Lamax_blood_ differs from the original *v*Lamax_muscle_, as it is normalized to 1 L of blood volume and is today measured as the maximal rate of blood lactate accumulation in mmol/L/s. To measure it, participants typically perform a 10–15-s all-out test followed by quantification of the rise of the blood lactate concentration from pre-test to the maximum after exercise. Some few seconds of a 10–15-s all-out test are “alactic” and should be subtracted from the work time to more accurately estimate the *v*Lamax_blood_. However, (1) glycolytic flux is unlikely to be truly maximal during an all-out exercise test, (2) peak glycolytic flux occurs only briefly, (3) there is no criterion for reaching the *v*Lamax_blood_, (4) there is no correction for lactate clearance in the time from exercise cessation to blood sampling, (5) there is no correction for ATP resynthesis by oxidative phosphorylation and (6) the *t*_alac_ correction is error-prone. Therefore, we propose the peak rate of lactate accumulation in mmol/L/s in arterialized earlobe or fingertip blood during an all-out exercise test lasting 10–15 s (*v*Lapeak) as a simplified estimate of peak glycolytic rate analogous to the $\dot{V}{\mathrm{O}}_{{2}}$ peak. In contrast to the *v*Lamax_blood_, the *v*Lapeak is not corrected for *t*_alac_. Modelling using Alois Mader’s model of human exercise metabolism suggests that (with everything else being the same) a higher *v*Lamax_muscle_ will (a) improve performance in events where a large part of the hydrolysed ATP is resynthesized by glycolysis, (b) cause a leftward shift of the lactate curve and (c) increase carbohydrate usage and accelerate glycogen depletion at a given exercise intensity. There is large potential for research on the validation and improvement of *v*Lapeak tests for athletes, healthy sedentary individuals and patients. This research should improve estimation of *v*Lamax_muscle_ from *v*Lapeak and experimentally test the modelling predictions of the effects of changes in *v*Lamax_muscle_ on exercise performance and fatigue.

## Key Points

**Table:** 

The *v*Lamax was introduced by Alois Mader in 1984 and defined as the “maximal rate of glycolysis expressed as rate of lactic acid formation”.
There are three variants of the *v*Lamax. First, there is the *v*Lamax_muscle_, which is in mmol/kg/s of lactate related to kilograms of active muscle mass and is used for metabolic modelling. Second, the *v*Lamax_blood_, introduced by Alois Mader as a measurable variant in 1994, is related to a litre of earlobe or fingertip blood and corrected for a so-called *t*_alac_. Third, to solve or simplify some issues with the *v*Lamax_blood_, we propose the *v*Lapeak as a variable analogous to the $\dot{V}{\mathrm{O}}_{{2}}$ peak; it is defined as the increase of the lactate concentration in earlobe or fingertip blood during a 10–15-s all-out test, without correction for *t*_alac_.
Metabolic modelling predicts that an increase of the *v*Lamax_muscle_ (or *v*Lamax_blood_ or *v*Lapeak, as all these variables correlate positively) will (a) increase glycolytic power and performance in events that are dependent on it, (b) cause a leftward shift of the power–lactate relationship and (c) increase carbohydrate and reduce fat utilization at a given work intensity.

## Introduction

In 1923, Archibald Vivian Hill and Hartley Lupton identified the maximal oxygen uptake ($\dot{V}{\mathrm{O}}_{{2}}$ max) [1], which today is defined as the “highest rate at which oxygen can be taken up and utilized by the body during severe exercise [at sea level]” [2]. The $\dot{V}{\mathrm{O}}_{{2}}$ max remains a physiological hallmark measurement. However, measuring a person’s $\dot{V}{\mathrm{O}}_{{2}}$ max only partially characterizes their energy metabolism. This is because the $\dot{V}{\mathrm{O}}_{{2}}$ max does not quantify glycolytic power (i.e. the peak rate of glycolytic ATP resynthesis) in working muscle. Information about glycolytic power is useful because glycolysis is the pathway that utilizes glucose and glycogen. Further, high glycolytic power, which is dependent on high concentrations of glycolytic enzymes in working muscle, is the basis for performing well in all-out bouts of exercise lasting from ~ 5 s to a few minutes. So, why is there no test specifically for glycolytic power, analogous to $\dot{V}{\mathrm{O}}_{{2}}$ max, our well-established gold standard measure of whole-body maximal oxidative phosphorylation?

Actually, a variable for glycolytic power was proposed over 40 years ago, and it is termed “*v*Lamax”. It is an odd variable; elite endurance athletes such as Belgian cyclist Wout van Aert talk about it [3], but there are only few relevant English-language publications, and few researchers seem to be aware of its origin. This is reflected by the title of a recent blog on the *v*Lamax,“Proper science or hocus pocus? Pros and cons of using the *v*Lamax” [4]. To add to the confusion, there are different versions of the *v*Lamax associated with different denominator units. One version is related to 1 kg of muscle, and a second to 1 L of blood, both of which were introduced by Alois Mader [5, 6]. On the basis of convention and to keep things clear and intuitive, we propose using the term “*v*Lamax_muscle_” for the version linked to active muscle mass and the term “*v*Lamax_blood_” for the version measured in a *v*Lamax test via blood lactate concentration change:

1. ***v*Lamax**_**muscle**_ (i.e. the original muscle *v*Lamax, also termed “dLa/dt_max_” and “$\dot{V}$_La.max_” [6] as well as “*v*_La,max_” first introduced in 1984 [7]). This is the (theoretical) maximal rate of glycolysis used by Alois Mader in his model of human energy metabolism. He defined it in 2003 as the “maximal rate of glycolysis expressed as rate of lactic acid formation, millimoles per second per kilogram” [6, 7]. Mader used *v*Lamax_muscle_ for modelling but never measured it.
2. ***v*Lamax**_**blood**_ (i.e. earlobe/fingertip blood or whole-body *v*Lamax; Mader originally called it “$\dot{V}$_La.max_” [5]). This variant describes the maximal rate of lactate accumulation in mmol/L per litre of earlobe or fingertip blood per second measured during an ~ 10–15-s all-out exercise test and corrected for a so-called alactic time (*t*_alac_), a correction for the contribution by the Lohmann reaction of 2–4 s. Alois Mader first proposed this variant in a 1994 book chapter where he discussed tests for glycolytic power [5].

**Fig. 1:**
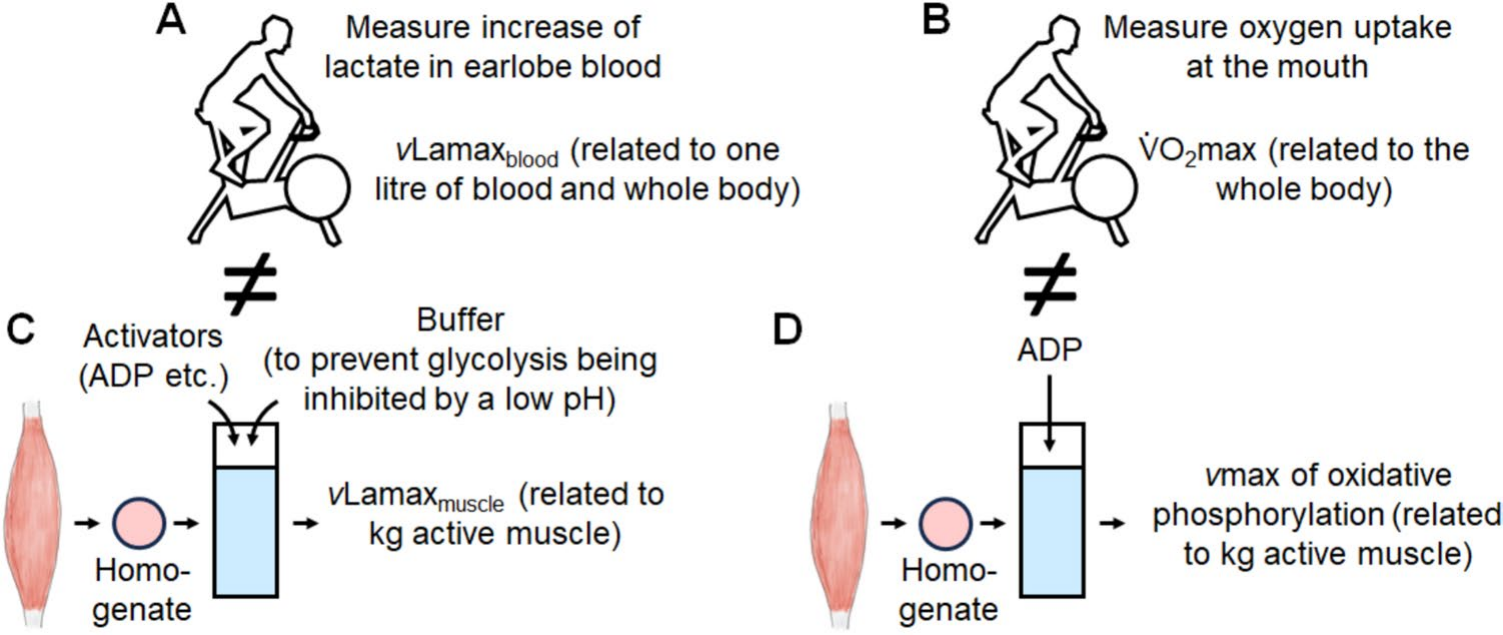
Analogy of the *v*Lamax and the $\dot{V}{\mathrm{O}}_{{2}}$ max. **A** The *v*Lamax_blood_ is the maximal rate of glycolysis measured as blood lactate accumulation per second during a suitable exercise test. **B** Similarly, the $\dot{V}{\mathrm{O}}_{{2}}$ max is the maximal rate of oxidative phosphorylation measured as oxygen uptake during a suitable exercise test at the whole-body level. **C** The *v*Lamax_blood_ is, however, different from the *v*Lamax_muscle_. In reality, it is impossible to measure whole glycolysis flux due to the integration of energy systems and the pyruvate dehydrogenase (PDH)-lactate dehydrogenase (LDH) complexes. **D** Similarly, the $\dot{V}{\mathrm{O}}_{{2}}$ max is not the *v*_max_ of oxidative phosphorylation of the active muscles extrapolated to the whole body, as the $\dot{V}{\mathrm{O}}_{{2}}$ max is primarily limited by oxygen transport, not the *v*_max_ of oxidative phosphorylation in muscle [8]

In summary, both the *v*Lamax_muscle_ and the *v*Lamax_blood_ represent the maximal rate of glycolysis or glycolytic power. However, their values differ in the same person, as the *v*Lamax_muscle_ is expressed per kilogram of active muscle mass, whereas the *v*Lamax_blood_ is expressed per litre of blood volume. The issue is similar for oxidative phosphorylation, as one could measure both the maximum velocity (*v*_max_) of oxidative phosphorylation of a muscle biopsy homogenate and the whole-body $\dot{V}{\mathrm{O}}_{{2}}$ max to characterize a person’s maximal oxidative phosphorylation. This is illustrated in Fig. 1.

In the remainder of this review, we will (1) introduce the maximal rate of lactate accumulation in mmol/L/s in arterialized earlobe or fingertip blood during an all-out exercise test lasting 10–15 s without correction for *t*_alac_ (*v*Lapeak) as a simplified variant that does not overpromise; (2) discuss how the *v*Lamax variants differ from, for example, the results of a Wingate test; (3) summarize publications on *v*Lamax; and (4) describe how a higher or lower *v*Lamax is predicted to affect exercise metabolism and performance.

## Measuring the ***v***Lamax_blood_ and Why We Propose the ***v***Lapeak

Measuring the $\dot{V}{\mathrm{O}}_{{2}}$ max during a graded exercise test is routine in human exercise laboratories. During a graded exercise test, the classic criterion that the participant has reached their $\dot{V}{\mathrm{O}}_{{2}}$ max is a plateau of the $\dot{V}{\mathrm{O}}_{{2}}$ despite increasing work rate [9, 10]. If the criteria for achieving $\dot{V}{\mathrm{O}}_{{2}}$ max during a test are not met, then the highest oxygen uptake observed is reported as $\dot{V}{\mathrm{O}}_{{2}}$ peak (see, e.g., Day et al. [11]). In contrast, there is currently no universally accepted protocol to measure the *v*Lamax_blood_, and there are numerous questions about how to conduct a *v*Lamax_blood_ test. In this section we will present *v*Lamax_blood_ testing, argue that it is impossible to directly measure the true *v*Lamax_blood_ and hence introduce the *v*Lapeak as a third, simplified variant of the *v*Lamax that does not overpromise with regards to the function that it measures.

After introducing the *v*Lamax_muscle_ as part of his model of human exercise metabolism in 1984 [6], Mader published ideas for a practical *v*Lamax_blood_ test in a 1994 book chapter [5]. In this chapter, he first critically evaluated glycolytic tests, such as 60–90-s all-out runs, where the running time and maximal post-exercise lactate were used as biomarkers for “anaerobic endurance”. To quantify the relative contribution of ATP resynthesis to performance in a 60–90-s run, he used his mathematical model of human energy metabolism to estimate the contributions of the metabolic pathways [6, 7]. His model predicted that, for a test where the athlete reaches task failure after 69 s, 32% of ATP provision is through phosphocreatine, 23% through oxidative phosphorylation and 45% through glycolysis. Thus, while glycolysis is the major contributor to ATP resynthesis, oxidative phosphorylation will cover nearly a quarter of ATP resynthesis. In other words, as a test of glycolytic power, an ~ 60-s test duration is too long to be valid because the rate of glycolysis will decline towards the end of the run due to the inhibitory effect of a low pH [12] and the aerobic power of an athlete will substantially contribute to test performance. He suggested that the accumulation of the lactate concentration during a 200 m run (~ 20 s in elite athletes) would be a better surrogate of glycolytic power if one corrected the result for ATP resynthesis via phosphocreatine, through a so-called *t*_alac_ correction [5].

On the basis of Mader’s reasoning, researchers today typically estimate the *v*Lamax_blood_ using an ~ 10–15-s all-out cycle ergometry or other exercise test [13–18]. During such a test, the investigator will measure the resting concentration of lactate and the highest concentration of lactate in the minutes after an all-out bout of exercise. The *v*Lamax_blood_ can then be calculated using the following formula:

1 $$v\mathrm{Lamax}_{\mathrm{blood}}= \frac{\mathrm{La}_{\mathrm{max}}- {\mathrm{La}}_{\mathrm{rest}}}{t_{\mathrm{exercise}}- t_{\mathrm{alac}}}$$

where *v*Lamax_blood_ (mmol/L/s) is the maximal rate of glycolysis expressed as the rate of lactic acid accumulation in earlobe or fingertip blood, La_max_ (mmol/L) is the maximal lactate concentration after exercise, La_rest_ (mmol/L) is the resting lactate concentration*, t*_exercise_ (s) is the duration of all-out exercise and *t*_alac_ (s) is the time equivalent of the estimated direct ATP resynthesis via phosphocreatine. This is typically assumed to be 3 s for a 10-s test [18] or is estimated individually as the time it takes for the mechanical power output to drop irreversibly by 3.5% from its peak in a Schoberer Rad Meßtechnik (SRM) cycle ergometry test [19]. The 3.5% value was chosen in part because the measurement tolerance of the SRM cycle ergometer is 2.5%, and so the reduction of 3.5% is assumed to be a true decline.

**Fig. 2:**
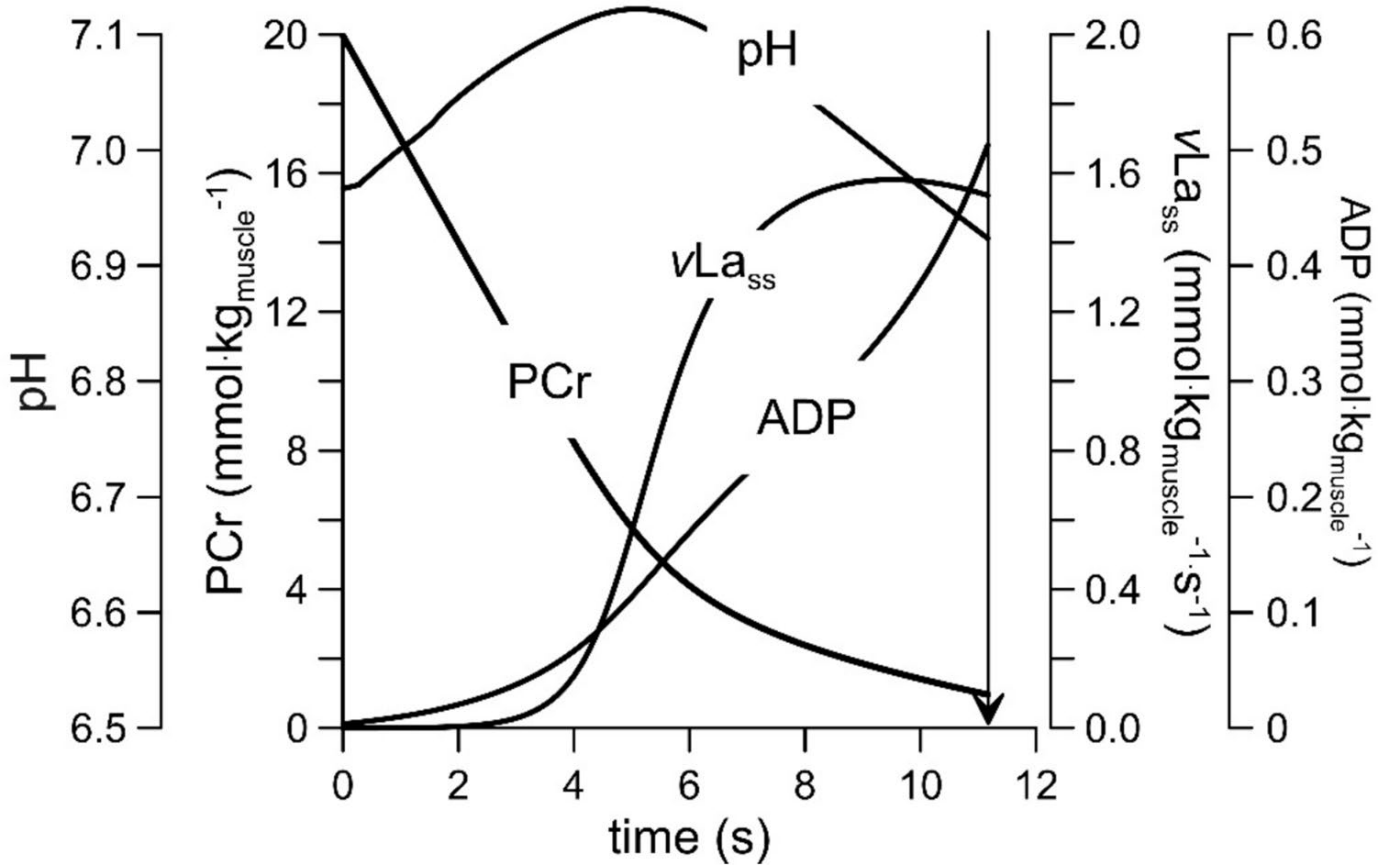
Simulation of energy metabolism during a ~ 11-s, 1300 W *v*Lamax_blood_ test as simulated with Alois Mader’s mathdematical model of human exercise metabolism [7, 20]. Note that the rate of lactate synthesis (*v*La_ss_) reaches its maximum only after ~ 6 s. It then plateaus even though ADP, a key activator [21], continues to increase. The reason is the drop of the pH from ~ 6 s onwards, as a low pH will inhibit phosphofructokinase [12]. Also note the slower decline of PCr at the point where the rate of lactate synthesis (*v*La_ss_) increases. If the rate of ATP hydrolysis is constant, then the concentration of PCr will drop less when the rate of glycolytic ATP resynthesis (i.e. *v*La_ss_) rises. Such behaviour is seen in ^31^P-NMR experiments [22]. The rise of the pH at the onset of exercise is caused by the rise of inorganic phosphate (Pi) buffer, as ATP is hydrolysed to ADP and Pi [23]. *PCr* phosphocreatine, *vLa*_*ss*_ the rate of lactate synthesis in mmol/kg_muscle_ /L/s

Unfortunately, there are six problems with the described *v*Lamax_blood_ test:

1. The intramuscular concentrations of ADP and AMP are predicted to rise throughout a *v*Lamax test (Fig. 2). It seems therefore unlikely that they reach concentrations required for maximal activation of phosphofructokinase and glycolysis. At the same time, once glycolytic flux increases from baseline, the pH will drop (Fig. 2), leading to an inhibition of phosphofructokinase and glycolysis. Thus, phosphofructokinase and glycolysis are unlikely to be fully activated (i.e. enzymes reaching their theoretically maximal rate) during all-out exercise in vivo.
2. Figure 2 also demonstrates that lactate synthesis (*v*La_ss_) reaches its in vivo peak during all-out exercise only for a fraction of a second before declining. Thus, measuring the increase of the lactate concentration during a 10–15-s test will underestimate the true maximal rate of lactate accumulation.
3. Even if a true *v*Lamax was reached, there is currently no criterion, such as the “levelling off” criterion in a $\dot{V}{\mathrm{O}}_{{2}}$ max test, that could inform the experimenter that the *v*Lamax has been reached.
4. The rise of the blood lactate concentration during a *v*Lamax_blood_ test is the result of lactate formation minus lactate clearance. There is currently no correction for lactate clearance.
5. Some ATP is resynthesized by oxidative phosphorylation. Even if this contribution is small, the calculation should be corrected for it.
6. The *t*_alac_ correction factor of 3 s for a 10-s test [18] or for the time from the start of the exercise to a drop from maximal power by 3.5% [19] is primarily based on metabolic modelling, and it likely varies between individuals, e.g., due to different phosphocreatine levels as a result of dietary strategies such as a vegan diet or creatine supplementation. Thus, the *t*_alac_ determination is error-prone.

Together, these six problems suggest that a *v*Lamax_blood_ test will not yield the true *v*Lamax_blood_, which is the rise of the blood lactate concentration per second if glycolysis was truly maximal in the active musculature and after *t*_alac_ correction for the person tested. To resolve this problem, we propose the *v*Lapeak as a third and simplified variant of the *v*Lamax. We do so, as we see no way to resolve the six problems with the *v*Lamax_blood_. This is based on precedent, as the $\dot{V}{\mathrm{O}}_{{2}}$ peak was introduced for similar reasons [11], and so it seems intuitive to use “peak” instead of “max” to denote that this is the measured peak rate but not necessarily the maximal possible rate.

Specifically, we define the *v*Lapeak as the peak rate of blood lactate accumulation in a 10–15-s all-out exercise test with no *t*_alac_ correction. The calculation is simple:

2 $$v{\mathrm{Lapeak}} = \frac{{{\mathrm{La}}_{{{\mathrm{max}}}} - {\mathrm{La}}_{{{\mathrm{rest}}}} }}{{t_{{{\mathrm{exercise}}}} }}$$

where *v*Lapeak (mmol/L/s) is the peak rate of glycolysis expressed as rate of lactic acid accumulation in arterialized (i.e. earlobe or fingertip) blood during an exercise test where glycolytic flux is near maximal (i.e. 10–15 s duration, depending on exercise mode), La_max_ (mmol/L) is the maximal arterialized blood lactate concentration in the minutes after exercise, and La_rest_ (mmol/L) is the resting arterialized blood lactate concentration.

Researchers can now develop *v*Lapeak tests for different exercise modes such as cycling, running, swimming and rowing. Generally, tests should be between 10 and 15 s long, but for example, in rowing a 20-s test has been used (Table 1, Table 2). Ideally, such tests can eventually be validated by correlating the *v*Lapeak values to *v*Lamax_muscle_ estimates obtained through metabolic simulation or estimated from a phosphofructokinase assay of a muscle biopsy. Moreover, such validation experiments can inform a conversion algorithm linking the *v*Lapeak with the *v*Lamax_muscle_, which is required for metabolic modelling with Alois Mader's model of human energy metabolism. As the duration of such a test is fixed, one could simplify even further and just report the rise of the arterialized blood lactate concentration from rest to the maximum after exercise (ΔLapeak) in an all-out 10–15-s test. However, calculating the *v*Lapeak is easy, and it allows for the comparison of data, e.g., of a 10-s to a 15-s test. This is why we recommend reporting the *v*Lapeak.

**Table 1:** Data from a systematic search for all PubMed publications that report *v*Lamax_blood_ data measured during maximal intensity tests with duration of ≤ 20 s

Participants (number and sex; age; fitness level)	Test protocol		*v*Lamax_blood_ (mmol/L/s)	*v*Lapeak (mmo/L/s)	References
	Duration and discipline	*t*_alac_			
13 M; 24.6 ± 4.8 years; various fitness levels	15 s cycling	Time to 3.5% power decrease	0.91 ± 0.18 (min: 0.67; max: 1.39)		[32]
17 M and 6 F; 26 ± 4 years; amateur cyclists	15 s cycling (performed thrice)	Time to 3.5% power decrease	1st test: 0.72 ± 0.13 (min: 0.37; max: 0.87) 2nd test: 0.72 ± 0.14 (min: 0.37; max: 0.87) 3rd test: 0.70 ± 0.14 (min: 0.35; max: 0.98)	1st test: 0.52 2nd test: 0.50 3rd test: 0.50	[33]
12 M; 26.0 ± 4.4 years; national-level competitive triathletes	15 s hand-cycling	Time to 3.5% power decrease	0.45 ± 0.11	0.37	[34]
32 M; 23.9 ± 2.4 years; trained individuals	Isokinetic test (performed twice) of 8 unilateral knee flexions and extensions	Time to 3.5% power decrease	1st test: 0.25 ± 0.11 2nd test: 0.27 ± 0.11	1st test: 0.21 2nd test: 0.21	[35]
30 M; SIT: 27.9 ± 1.8/ET: 26.7 ± 2.2/c: 27.5 ± 1.7 years; physical education students	15 s cycling (repeated every 2 weeks; 6-week intervention)	Time to 3.5% power decrease	Baseline: 0.76 ± 0.18/ 0.75 ± 0.18/ 0.75 ± 0.18/ 2 weeks: 0.67 ± 0.17/ 0.76 ± 0.18/ 0.75 ± 0.18/ 4 weeks: 0.63 ± 0.17/ 0.74 ± 0.17/ 0.73 ± 0.18 6 weeks: 0.63 ± 0.15/ 0.74 ± 0.17/ 0.75 ± 0.21	Baseline: 0.53/ 0.53/ 0.55/ 2 weeks: 0.46/ 0.55/ 0.57/ 4 weeks: 0.45/ 0.53/ 0.54/ 6 weeks: 0.44/ 0.52/ 0.53	[36]
11 M and 5 F; 23.1 ± 2.9 years; competitive runners	100 m running (performed 3 times), with three different measurement methods; mean 100-m time ranged from 13.78 to 13.90 s	Estimated through interpolation from 3.5% power output decrease from maximal and 3–4-s estimates	Method 1: 0.79 ± 0.18 0.78 ± 0.21 0.76 ± 0.20 Method 2: 0.79 ± 0.18 0.76 ± 0.18 0.75 ± 0.18 Method 3: 0.80 ± 0.18 0.77 ± 0.18 0.75 ± 0.18	Method 2: 0.59 0.57 0.56 Method 3: 0.60 0.57 0.56	[37]
14 M in the HVLL group and 10 M in the LLHL group; HVLL 25.0 ± 4.3 years/LLHL 24.6 ± 2.8 years; all strength-trained	Concentric isokinetic strength test of 10 thigh extensions and flexions (15 s) repeated twice, pre-and post-6-week resistance training intervention	3 s	HVLL: Pre: 0.271 ± 0.067 Post: 0.298 ± 0.067 LVHL: Pre: 0.249 ± 0.122 Post: 0.291 ± 0.089	HVLL: Pre: 0.23 Post: 0.25 LVHL: Pre: 0.18 Post: 0.24	[38]
15 M and 3 F; 25.1 ± 2.8 years; competitive triathletes	15 s cycling and hand-cycling, performed twice	Time until peak power output was reached	Cycling: 0.53 ± 0.14 and 0.52 ± 0.14 Hand-cycling: 0.31 ± 0.09 and 0.32 ± 0.10		[39]
29 M and 15 F; 25.4 ± 4.1 years; trained runners and triathletes	100 m running (13.14 ± 0.58 s and 15.39 ± 1.14)	time_100_ × 0.0909 + 2.0455	M: 0.74 ± 0.14 F: 0.55 ± 0.13 Total: 0.67 ± 0.16	M: 0.55 F: 0.42 Total: 0.50	[17]
15 M and 3 F; 25.1 ± 2.8 years; competitive triathletes	100 m running (13.50 ± 1.23 s and 13.41 ± 1.26 s) and 15 s cycling (performed twice)	Time to 3.5% power decrease	Running: 0.73 ± 0.16 and 0.71 ± 0.16 Cycling: 0.60 ± 0.16 and 0.60 ± 0.15	Running: 0.53 and 0.51 Cycling: 0.41 and 0.42	[40]
30 M; 30 ± 6 years; national-level track cyclists	15 s cycling	Three calculations: 1. When power output decreased by 3.5% from maximal 2. Time until peak power output was reached 3. Time until peak power output was reached plus time attributed to calculated oxidative contribution	Calculation 1: 0.97 ± 0.18 Calculation 2: 0.85 ± 0.12 Calculation 3: 0.88 ± 0.13	0.78	[41]
7 M and 3 F; 19.8 ± 0.9 years; national rowers	10 s rowing (ergometer)	*t*_exer_ × 0.0909 + 2.0455	0.25–0.66		[42]
8 M and 6 F; M: 21.7 ± 4.8 years/F: 18.2 ± 3.0 years; highly trained swimmers	25 m and 35 m swimming (25 m: 11.75 ± 1.38 s/35 m: 17.76 ± 2.04 s)	25 m: 3.5 s 35 m: 4 s	25 m: 0.75 ± 0.18 35 m: 0.54 ± 0.18	25 m: 0.51 35 m: 0.41	[16]
25 M; 27 ± 6 years; trained individuals	10 s and 15 s cycling	Time to 3.5% power decrease	10 s: 0.86 ± 0.17 15 s: 0.68 ± 0.18		[13, 43]
30 M and 20 F; 31.2 ± 7.8 years; cyclists	15 s cycling (performed twice)	Three calculations: 1. 3.5 s 2. Time until peak power output was reached 3: When power output decreased by 3.5% from maximal	Calculation 1: 0.55 ± 0.14 and 0.54 ± 0.13 Calculation 2: 0.50 ± 0.11 and 0.49 ± 0.13 Calculation 3: 0.56 ± 0.13 and 0.53 ± 0.14	0.42 and 0.42	[14]
19 M and 10 F; M: 28.3 ± 4.4 years/F: 25.3 ± 2.5 years; cyclists of various levels	15 s cycling	Time to 3.5% power decrease	M: 0.59 ± 0.15 F: 0.50 ± 0.15		[44]
17 M; 19.9 ± 1.7 years; well-trained swimmers	20 m swimming (11.5 ± 0.4 s)	3 s	0.63 ± 0.14	0.46	[45]
9 M and 8 F; M: 21.3 ± 6.7 years/F: 17.7 ± 2.2 years; trained rowers	20 s rowing (performed twice)	4 s	Day 1: 0.29 ± 0.11 Day 2: 0.28 ± 0.10		[46]
19 M and 15 F; M: 19.2 ± 3.3 years/F: 17.9 ± 1.7 years; runners (specializing in 100, 400 and 800 m)	100 m running (11.86 ± 0.46 s and 13.43 ± 0.81 s)	Time to reach maximal power output	M: 0.88 ± 0.19 F: 0.73 ± 0.13		[47]
13 M and 8 F; 23.1 ± 1.9 years; sport university students	15 s running (performed 5 times under different conditions)	*t*_exer_ × 0.0909 + 2.0455	0.59 ± 0.09; 0.51 ± 0.01; 0.53 ± 0.1; 0.54 ± 0.1; 0.57 ± 0.1	0.46; 0.40; 0.41; 0.42; 0.44	[15]
13 M; 25.0 ± 2.4 years; trained individuals	10 s cycling at 5 different pedalling frequencies	3 s	90 rpm: 0.63 ± 0.14 110 rpm: 0.76 ± 0.13 130 rpm: 0.86 ± 0.16 150 rpm: 0.88 ± 0.15 170 rpm: 0.94 ± 0.14	90 rpm: 0.44 110 rpm: 0.54 130 rpm: 0.60 150 rpm: 0.61 170 rpm: 0.66	[48]

*M* males, *F* females, *SIT* sprint-interval training, *ET* endurance training, *c* control, *HVLL* high-volume low-load, *LLHL* low-volume high-load

**Table 2:** Case study of three world-class rowers on rowing ergometers

Rower	Average power over 2000 m	Power at 4 mmol/L lactate	*v*Lamax_blood_^a^ (20-s rowing test)	Peak lactate after 2000 m test
A	495 W	295 W	0.48 mmol/L/s	22.7 mmol/L
B	492 W	331 W	0.32 mmol/L/s	18.3 mmol/L
C	495 W	372 W	0.19 mmol/L/s	15.3 mmol/L

^a^*v*Lamax_blood_ values tend to be lower during rowing than during, e.g., cycling. The likely reason is that there is reduced muscle activation during the recovery phase after the drive phase of the stroke in key muscles during rowing

## What Factors Influence the ***v***Lamax_muscle_?

The *v*_max_ of a chemical reaction (the *v*Lamax_muscle_ reflects the *v*_max_ for glycolysis) is directly proportional to the concentration of the enzyme that catalyses the reaction [24]. Glycolysis is a multi-enzyme sequence of chemical reactions, but this sequence has a single rate-limiting enzyme, phosphofructokinase (PFK) [12, 24]. Critically, the cytosolic concentration of PFK differs substantially across muscle fibres and motor units. For example, Murgia et al. (2021), using single muscle fibre proteomics, showed that vastus lateralis muscle fibres express four phosphofructokinase-1 protein isoforms (abbreviated as PFKM, PFKL, PFKFB2 and PFDFB3) of which the concentration of PKFM (“M” indicates “muscle”) was by far the highest. It was 322 ± 301 in arbitrary units (AU) in type 1, 2067 ± 1935 AU in type 2A muscle fibres and 2285 ± 1724 AU in type 2X muscle fibres. This translated to an ~ sevenfold variation in PFKM from type 1 to type 2X muscle fibres in the 22–27-year-old men investigated [25]. Thus, the muscle fibre distribution and specifically a high percentage and large size of type 2X and type 2A fibres in the exercising muscles will correlate with a high *v*Lamax_muscle_ (and hence *v*Lamax_blood_ and *v*Lapeak, as all versions of the *v*Lamax should correlate) for that exercise mode. As *v*Lamax_blood_ is a whole-body variable, a higher percentage of leg muscle (lean) mass in relation to total body mass will further increase the *v*Lamax. This is consistent with the observation that a high lean lower body mass predicts performance in a Wingate test [26].

## What is the Difference Between the ***v***Lapeak and Results of Other “Anaerobic” Tests?

One possible critique of *v*Lapeak or *v*Lamax_blood_ tests is that there are already many “anaerobic” (here, “anaerobic” refers to an ATP-generating energy metabolism pathway that is not reliant on oxygen) tests [27]. The best known of these tests is the Wingate test, where participants pedal on a cycle ergometer for 30 s all out against a constant resistance that is scaled to bodyweight [28]. During such a test, elite and sub-elite 400 m sprinters have been reported to reach maximal post-exercise lactate concentrations of 19.1 ± 2.4 and 17.5 ± 2.8 mmol/L, respectively [29]. What is the added value of specifically quantifying *v*Lapeak compared with data generated by performing traditional anaerobic tests? There are two differences. First, a valid *v*Lapeak test will measure lactate as the key product of glycolysis, whereas for example, the Wingate test measures non-metabolic variables such as peak power and a fatigue index. These variables are dependent not only on the *v*Lamax_muscle_ but also on other variables, including the phosphocreatine concentration at rest, ATP resynthesis by oxidative phosphorylation and leg muscle mass [26]. Second, using the *v*Lapeak to estimate the *v*Lamax_muscle_ is a strategy to “feed” Alois Mader’s model of human exercise metabolism or other metabolic models [6, 7, 30]. Mader’s model is a mechanistic, 33-equation model of human energy metabolism that allows simulating the response of key metabolic variables such as blood lactate and the $\dot{V}{\mathrm{O}}_{{2}}$ to a bout of exercise in a metabolically defined individual [7].

## What do ***v***Lamax Publications Report?

For the $\dot{V}{\mathrm{O}}_{{2}}$ max, there are many reference data for women and men at different ages, both trained and untrained. For example, already in 1963, Wildor Hollmann had plotted the $\dot{V}{\mathrm{O}}_{{2}}$ max data of 2834 women and men across an age range of 6–80 years [31]. What about the *v*Lamax_blood_? To give an overview of published *v*Lamax_blood_ values, we systematically searched PubMed for publications that contained data on the *v*Lamax_blood_ obtained during all-out tests lasting ≤ 20 s, using the keyword “*v*Lamax” and keywords (“lactate accumulation rate” OR “lactate formation rate” OR “lactate production rate” OR “glycolytic rate” AND “exercise”), as well as variations of the terms in quotes. Table 1 summarizes the results of these tests. We additionally estimated the *v*Lapeak wherever the reported data allowed it.

Table 1 suggests that *v*Lamax_blood_ values typically range from ~ 0.2 to 1.0 mmol/L/s, with 0.2 mmol/L/s being low glycolytic (“slow twitcher”) and 1.0 mmol/L/s being a highly glycolytic athlete (“fast twitcher”). The values of the suggested *v*Lapeak will be lower, as there is no correction for the *t*_alac_, so the change of lactate is divided by more seconds. The *v*Lapeak values that we calculated from the published *v*Lamax_blood_ values ranged from ~ 0.2 to 0.8 mmol/L/s and were on average 28% lower than the values for the *v*Lamax_blood_.

As *v*Lamax_blood_ tests require a maximal effort, there are questions about test–retest reliability. Here, *v*Lamax_blood_ reliability studies have reported good to excellent test–retest reliability, suggesting that *v*Lamax_blood_ or *v*Lapeak tests are reliable [14, 33, 37, 39, 40, 45, 46].

## A Higher ***v***Lamax is Predicted to Left-Shift the Lactate-Performance Curve

**Fig. 3:**
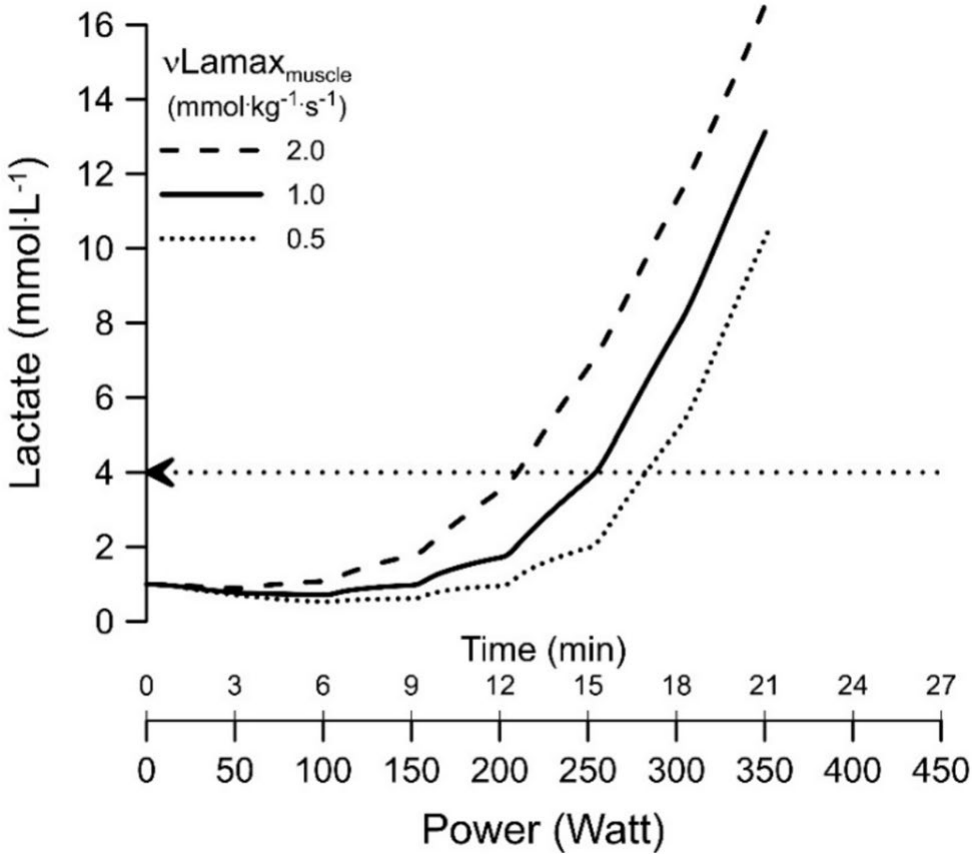
Mader model simulation of the effect of variations of the *v*Lamax_muscle_ on the power–lactate curve. The simulation of the lactate–power relationship assumes a 70 kg person with 28% active muscles and a $\dot{V}{\mathrm{O}}_{{2}}$ max of 56 mL/min/kg with all other variables being identical. The *v*Lamax_muscle_ values are 2.0 mmol/kg/s (glycolytic athlete), 1.0 mmol/kg/s (average *v*Lamax_muscle_ athlete) and 0.5 mmol/kg/s (low-glycolytic athlete). The example is from Heck et al. [20]. The *v*Lamax_muscle_ values used in this simulation correspond to *v*Lamax_blood_ values of 0.9, 0.45 and 0.25 mmol/L/s, respectively

In the last part of this review, we will discuss predictions obtained with Alois Mader’s model of human exercise metabolism [6, 7, 49]. One might assume that the ideal endurance athlete will combine a high $\dot{V}{\mathrm{O}}_{{2}}$ max and mitochondrial content with a high *v*Lamax_muscle_, as their summated “maximal ATP resynthesis rate” would be highest. One might assume that such an athlete would combine high endurance with an additional ability of doing well during glycolytic high-intensity periods. However, simulations with Mader’s model of human exercise metabolism [6, 7] suggest that this is a misconception because a high *v*Lamax will cause a leftward shift of the lactate–power curve due to higher lactate synthesis rates. The predicted effect of variations of the *v*Lamax_muscle_ on the lactate–power curve with other variables such as the $\dot{V}{\mathrm{O}}_{{2}}$ max being identical is illustrated in Fig. 3.

**Fig. 4:**
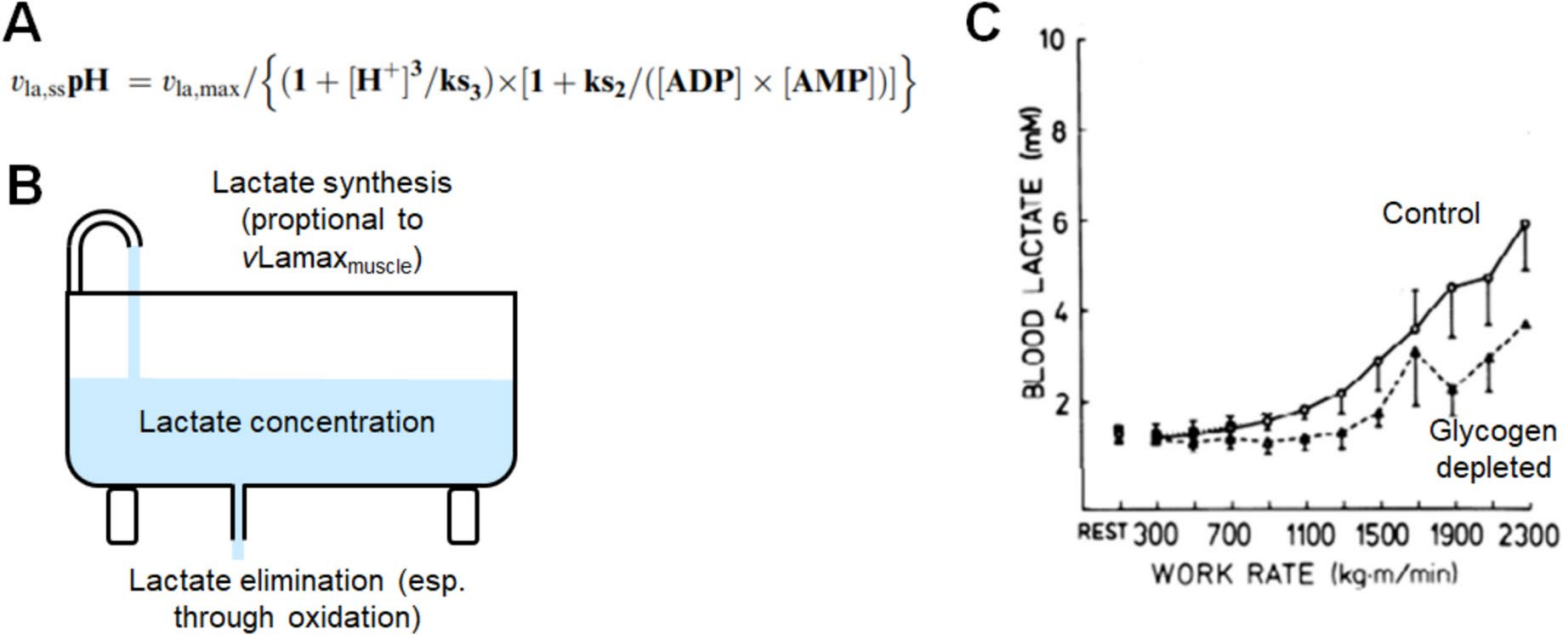
**A** Mader’s formula that includes the *v*Lamax_muscle_, which he stated as *v*_la,max_. Briefly, the formula allows for the calculation of glycolytic flux or the rate of lactate formation (*v*_La,ss,_pH). In the equation, “ks_2_” and “ks_3_” are constants, ADP and AMP are activators of glycolysis and H^+^ is an inhibitor, reflecting the fact that a low pH inhibits phosphofructokinase [7, 12]. **B** A simple schematic illustrating how the concentration of lactate in blood will be affected by lactate synthesis and lactate elimination primarily by oxidative phosphorylation. As lactate synthesis is proportional to the *v*Lamax_muscle_, a 20% higher or lower *v*Lamax_muscle_ will increase or decrease lactate synthesis by 20% as well. **C** Indirect evidence for the effect of a lower *v*Lamax_muscle_ on the lactate–power relationship [53]. A carbohydrate-low diet has an effect comparable to the reduction of the *v*Lamax_muscle_ (or *v*Lapeak [15]) and causes a rightward shift of the lactate–power curve [53] presumably because of a decreased rate of lactate synthesis. Note that we have removed a third curve from the original figure so that the figure only shows the data relevant to this discussion

The reasons for the effect of the *v*Lamax_muscle_ on the lactate–power relationship are illustrated in Fig. 4B, and experimental evidence for the effect of a lower *v*Lamax_muscle_ on the lactate–power relationship is shown in Fig. 4C. Figure 4A reproduces Mader’s equation from his 2003 publication where the *v*Lamax_muscle_ features [7].

The blood lactate concentration depends on the rate of lactate synthesis minus lactate clearance primarily via oxidative phosphorylation [50, 51]. As lactate synthesis scales linearly with the *v*Lamax_muscle_, an arbitrary 20% higher *v*Lamax_muscle_ will result in a 20% higher rate of lactate synthesis with everything else being the same. Conversely, if the *v*Lamax_muscle_ is reduced by 20%, then this will lower the rate of lactate synthesis by 20% and cause a rightward shift of the power-lactate relationship. There is indirect experimental evidence for this prediction. Carbohydrate restriction has an effect comparable to a reduction of the *v*Lamax_muscle_ (or *v*Lapeak [15, 52]) and causes, as predicted, a rightward shift of the power–lactate curve (Fig. 4C [53]).

The insight that a high *v*Lamax_muscle_ indicates a higher maximal rate of lactate synthesis and thus a higher concentration of lactate at a given workload has implications for endurance athletes. In endurance sports with constant pacing (e.g. marathon running, cycling time trials or triathlon), a low *v*Lamax_muscle_ is predicted to allow the athlete to race at a higher velocity at a given lactate concentration when compared with an athlete with a higher *v*Lamax_muscle_. For this reason, athletes in constant-intensity endurance sports should aim to increase their mitochondrial density in their working muscles to increase lactate clearance through oxidative phosphorylation [50, 51] while lowering their *v*Lamax_muscle_ (albeit not through glycogen depletion, as this reduces performance).

More intermittent endurance sports, however, require not a high but a suitable *v*Lamax_muscle_ for that sport. These are sports where the intensity varies for tactical reasons (e.g. championship 10 km runs and road cycling), due to the intermittent nature of the sport (soccer) or because of the course topography (e.g. Nordic skiing or cyclocross with high-intensity phases uphill followed by low-intensity downhill phases). If increased glycolytic activity is required for high-intensity phases, then athletes should seek to raise their *v*Lamax_muscle_. However, if the *v*Lamax_muscle_ is too high, then the athlete might do well during high-intensity phases and sprints but will struggle to last the distance, as they will have higher lactate concentrations and use more carbohydrate and glycogen-deplete earlier than a low-*v*Lamax_muscle_ athlete with all other variables being identical.

## A Higher ***v***Lamax Increases Carbohydrate Usage and thus Accelerates Glycogen Depletion and Reduces Fat Oxidation

**Fig. 5:**
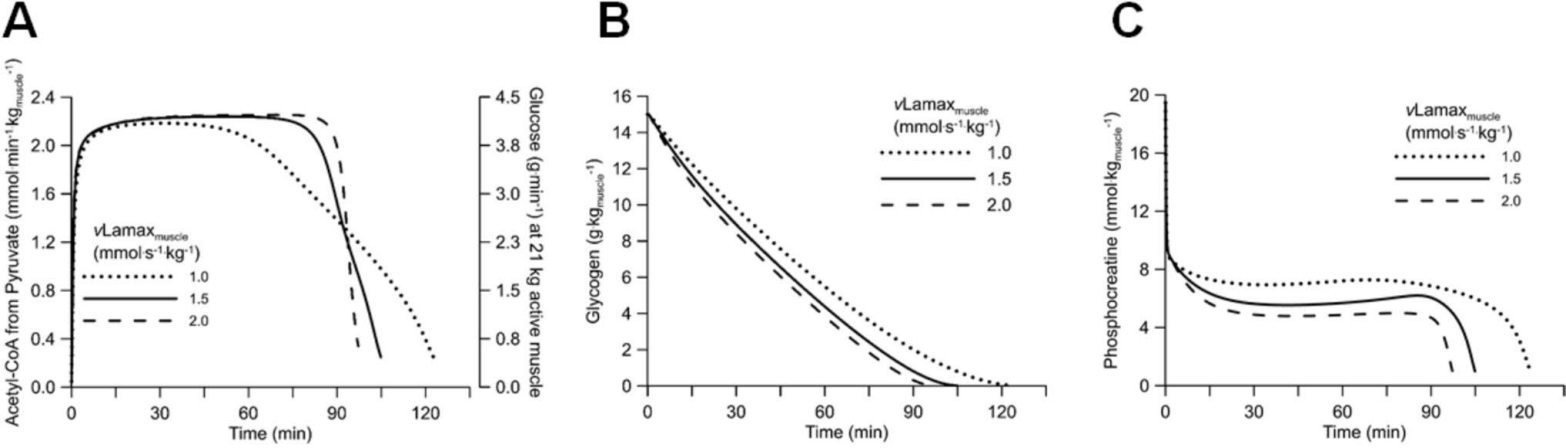
Glycogen during a simulated 275 W exercise in three athletes with a $\dot{V}{\mathrm{O}}_{{2}}$ max of 60 L/min/kg, 15 g glycogen per kg muscle and *v*Lamax_muscle_ values of 2.0, 1.5 and 1.0 mmol/kg/s. Fatigue is estimated to occur when the concentration of phosphocreatine declines to below 1 mmol/kg. **A** Acetyl-CoA from pyruvate (i.e., carbohydrate oxidation), **B** concentration of glycogen, and **C** concentration of phosphocreatine

With other variables being the same, an athlete with a higher *v*Lamax_muscle_ is predicted to utilize more carbohydrates (i.e. glucose and glycogen) and thus glycogen-deplete more rapidly during exercise than an athlete that has a lower *v*Lamax_muscle_. This is illustrated in Fig. 5 for three athletes that cycle at 275 W with a $\dot{V}{\mathrm{O}}_{{2}}$ max of 60 L/min/kg, 15 g stored glycogen per kg muscle and *v*Lamax_muscle_ (i.e. not *v*Lamax_blood_) values of 2.0, 1.5 and 1.0 mmol/kg/s, respectively. The simulation with Mader’s model suggests that the athlete with a *v*Lamax_muscle_ of 2.0 mmol/kg/s is predicted to fatigue after 97 min due to glycogen depletion, versus 105 min with a *v*Lamax_muscle_ of 1.5 mmol/kg/s and 124 min with a *v*Lamax_muscle_ of 1.0 mmol/kg/s. Fatigue is estimated to occur when the concentration of phosphocreatine declines to below 1 mmol/kg.

Assuming that the predictions derived from Mader’s model simulations are supported by experimental data, his model predicts that athletes with a higher *v*Lamax_muscle_ fatigue more than 20 min earlier during exercise lasting ~ 2 h to fatigue, as they will glycogen-deplete earlier. However, athletes can delay glycogen depletion by carbohydrate feeding. Thus, another prediction is that athletes with a high *v*Lamax_muscle_ should ingest more carbohydrates during a given long-duration exercise, as they will use more glucose/glycogen (in the simulation, especially between 30 and 90 min). These predictions can and should be experimentally confirmed.

## Rowing Case Study

Finally, we discuss three world-class rowers who all managed to generate an average power of ~ 495 W during a 2000 m rowing competition test on a Concept2 ergometer. An average power of 495 W translates to a 2000 m rowing time of ~ 5:56. The rowers additionally performed a 3 × 8 min test on the rowing ergometer to estimate the power at 4 mmol/L lactate [54] and additionally measured the *v*Lamax_blood_ during a 20-s all-out rowing test. The main finding of the case study is that the rowers achieved an almost identical 2000 m Concept2 ergometer performance time despite their power at 4 mmol/L of lactate differing by 77 W between rowers A and C. However, the *v*Lamax_blood_ test indicates that rower A is the most glycolytic rower followed by B and then C, suggesting that rower A was compensating with a high glycolytic power (i.e. *v*Lamax_blood_) for his low power at 4 mmol/L of lactate. This is further supported by the peak lactate concentrations after the 2000 m test. Thus, rower A achieves the 2000 m performance with a higher glycolytic contribution than rowers B and C, whereas rower C relies more on oxidative phosphorylation than rowers A and B (Table 2).

Their different metabolic profiles also have implications for training. For example, if these athletes would train intervals with 350 W, rower C would be below 4 mmol/L of lactate concentration and should recover quickly after an interval. In contrast, rower A would be above 4 mmol/L and the concentration of blood lactate would increase with the duration of the interval, causing the pH to drop and inhibit glycolysis. Thus, while rower C would be expected to recover quickly after an interval, rower A would take more time to normalize his pH so that glycolysis is not inhibited at the start of the following interval. This case study highlights the usefulness of measuring the *v*Lapeak for metabolic profiling of athletes. Despite their similar maximal 2000 m ergometer test results, the athletes would likely benefit from individualized interval training prescriptions.

## Summary and Future Research

*v*Lapeak is the peak rate of glycolysis measured as the maximal rise of the lactate concentration in mmol/L/s at the whole-body level during an 10–15-s all-out test without a *t*_alac_ correction. Using this definition, the *v*Lapeak is for glycolysis what the $\dot{V}{\mathrm{O}}_{{2}}$ peak is for oxidative phosphorylation, namely a whole-body measure of a peak rate of a major metabolic pathway. A *v*Lapeak test is not just another anaerobic test, such as the Wingate test, as it specifically measures the maximal rate of glycolysis and not performance variables that are only partially influenced by a high glycolytic power.

The *v*Lapeak has implications not only for performance as discussed earlier but also potentially for our metabolic health, as glycolysis is the main pathway of glucose disposal. On this basis, we argue that exercise physiologists should investigate the *v*Lapeak to fill the many gaps in our knowledge. Important questions for future research include:

1. How accurate, valid and reliable are *v*Lapeak tests for different exercise modes? What test criteria (e.g. no glycogen depletion) help ensure a valid test?
2. Is it possible, safe and informative to estimate the *v*Lapeak in non-athlete populations such as patients with metabolic disease and elderly individuals?
3. How well do the results of a *v*Lapeak test correlate with the *v*Lamax_muscle_, perhaps estimated through a phosphofructokinase enzyme assay of a muscle homogenate of the exercising muscles? This question should be answered through muscle-biopsy-based validation experiments.
4. Is it possible to develop indirect and possibly non-invasive tests where we convert the result of that test into a *v*Lapeak_muscle_ estimate for that exercise mode? This would be similar to doing a shuttle test to estimate the $\dot{V}{\mathrm{O}}_{{2}}$ max [55].
5. Can the predictions of Mader’s model of human energy metabolism (i.e. a high *v*Lamax will cause a left-shift of the power-lactate curve and increase carbohydrate oxidation) be validated experimentally?
6. Due to ramp-like recruitment, athletes will exercise with few active, highly glycolytic type 2 muscle fibres at low intensity and only recruit the majority of type 2 muscle fibres during high-intensity exercise [56]. Can this be incorporated into Mader's model of human energy metabolism?
7. What types of exercise training (e.g. sprint interval training, resistance training, hypoxic exercise) and what other interventions (e.g. bicarbonate supplementation) can increase or decrease the *v*Lapeak, keeping in mind that exercise training increases lactate oxidation most [50, 51]?
8. How do exercise stimuli, hypoxia and other stimuli regulate the expression of glycolytic enzymes such as phosphofructokinase? What are the signal transduction mechanisms that link these stimuli to the transcription and translation of glycolytic enzyme genes? While the stimulation of mitochondrial biogenesis by exercise through factors such as PGC-1α [57] is well documented, we poorly understand the adaptation of glycolysis to exercise and other interventions.

We look forward to future research into the *v*Lamax_muscle_ and *v*Lapeak and its implications for human health and performance.

## Declarations

### Availability of data

We will make the simulation data available in the case of a reasonable request.
